# The association of care burden with motivation of vaccine acceptance among caregivers of stroke patients during the COVID-19 pandemic: mediating roles of problematic social media use, worry, and fear

**DOI:** 10.1186/s40359-023-01186-3

**Published:** 2023-05-15

**Authors:** Shikha Kukreti, Carol Strong, Jung-Sheng Chen, Yi-Jung Chen, Mark D. Griffiths, Meng-Tsang Hsieh, Chung-Ying Lin

**Affiliations:** 1 Department of Public Health, College of Medicine, National Cheng Kung University, Tainan, Taiwan; 2 Department of Medical Research, E-Da Hospital, Kaohsiung, 82445 Taiwan; 3 Institute of Allied Health Sciences, National Cheng Kung University Hospital, College of Medicine, National Cheng Kung University, 1 University Rd, Tainan, 701 Taiwan; 4 International Gaming Research Unit, Psychology Department, Nottingham Trent University, Nottingham, NG1 4FQ UK; 5 Stroke Center and Department of Neurology, E-Da Hospital, I-Shou University, Kaohsiung, 82445 Taiwan; 6 School of Medicine, College of Medicine, I-Shou University, Kaohsiung, 82445 Taiwan; 7 Institute of Clinical Medicine, College of Medicine, National Cheng Kung University, Tainan, 70101 Taiwan; 8 Biostatistics Consulting Center, National Cheng Kung University Hospital, College of Medicine, National Cheng Kung University, Tainan, 70101 Taiwan; 9 Department of Occupational Therapy, College of Medicine, National Cheng Kung University, Tainan, 70101 Taiwan

**Keywords:** Care burden, Stroke, COVID-19 vaccine acceptance, Problematic social media use

## Abstract

**Background:**

The aim of the present study was to investigate the relationship between care burden and motivation of COVID-19 vaccine acceptance among caregivers of patients who have experienced a stroke and to explore the mediating roles of social media use, fear of COVID-19, and worries about infection in this relationship.

**Methods:**

A cross-sectional survey study with 172 caregivers of patients who had experienced a stroke took part in a Taiwan community hospital. All participants completed the Zarit Burden Interview, Bergen Social Media Addiction Scale, Worry of Infection Scale, Fear of COVID-19 Scale, and Motors of COVID-19 Vaccine Acceptance Scale. Multiple linear regression model was applied to construct and explain the association among the variables. Hayes Process Macro (Models 4 and 6) was used to explain the mediation effects.

**Results:**

The proposed model significantly explained the direct association of care burden with motivation of COVID-19 vaccine acceptance. Despite the increased care burden associated with decreased vaccine acceptance, problematic social media use positively mediated this association. Moreover, problematic social media use had sequential mediating effects together with worry of infection or fear of COVID-19 in the association between care burden and motivation of vaccine acceptance. Care burden was associated with motivation of vaccine acceptance through problematic social media use followed by worry of infection.

**Conclusions:**

Increased care burden among caregivers of patients who have experienced a stroke may lead to lower COVID-19 vaccines acceptance. Moreover, problematic social media use was positively associated with their motivation to get COVID-19 vaccinated. Therefore, health experts and practitioners should actively disseminate accurate and trustworthy factual information regarding COVID-19, while taking care of the psychological problems among caregivers of patients who have experienced a stroke.

## Introduction

The coronavirus disease 2019 (COVID-19) has had a profound impact on individuals and society globally [1, 2]. Cardiovascular risk factors, including hypertension, coronary heart disease, heart failure, and ischemic stroke are common among patients with COVID-19 infection and are associated with mortality [3, 4]. The COVID-19 pandemic and associated lockdowns also appear to have negatively impacted the lives of vulnerable populations, including individuals experiencing strokes and their caregivers [5, 6]. The COVID-19 pandemic profoundly impacted healthcare for stroke patients as well as follow-up care [7, 8].

One method to minimize the COVID-19 impacts among stroke patients is vaccination. COVID-19 vaccination is an important part of COVID-19 management strategy for governments and health officials. For the vaccine to be effective, it is important to ensure that the population is generally compliant with the vaccination program. Subsequently, high-risk population such as patients who have experienced strokes need to have high uptake of the vaccine and the issue of any ‘vaccine hesitancy’ in this population needs to be addressed. Previous studies have explored the views of the COVID-19 vaccine from individuals who have experienced a stroke [9, 10]. However, there is a gap in the literature regarding the perceptions of caregivers of stroke patients towards the COVID-19 vaccine.

One key issue regarding the perceptions of caregivers of stroke patients toward the COVID-19 vaccine is their care burden. In contrast to degenerative diseases such as dementia, a stroke is an acute event, so families are faced with immediate and unexpected care demands. Studies indicated that caregivers' quality of life is affected during the treatment course of a patient [11, 12]. The literature shows that caregivers for individuals who have had a stroke suffer from numerous overwhelming care burdens that can be both physical (e.g., fatigue) [13, 14] and psychological (e.g., depression and anxiety) [15]. These types of care burdens have been particularly prevalent among caregivers who live with their care recipients and provide care for long periods of time [16]. However, there might be differences in care burdens among caregivers based on how they are affected. The importance of this knowledge in tailoring interventions to support caregivers in times of crisis has been understudied.

Caregiver users who use social media to share their everyday practices may also be at risk for problematic social media use. Previous research on problematic social media use has shown that normal use of social media can reduce feeling of loneliness and foster the experience of social support [17] whereas maladaptive use can contribute to addictive tendencies and negatively impact mental health [18, 19]. Exposure to vaccine-related misinformation exchanged on social media has previously been found to influence vaccine acceptance rates [20]. Moreover, recent research applying the Health Belief Model suggested that vaccine intention was influenced by what others are doing and information available to them on social media sites [21]. Therefore, it was posited that problematic social media use would be a mediator in the relationship between care burden and motivation of COVID-19 vaccine acceptance. Despite the clear benefits of vaccines [22], caregivers need to be assured that getting COVID-19 vaccination is beneficial for their own health to provide quality care to the person they care for.

Fear of COVID-19 and its impacts, including worries of infection have been among the major psychosocial stresses during the pandemic [23]. In addition, problematic social media use may result from prolonged exposure to social media and/or overdependence on it for information. Problematic social media use has been associated with worries of infection [24] and has also been found to influence fear of COVID-19 [25]. However, there is still a gap in the literature related to how problematic social media use, fear of COVID-19, and worry of infection mediate the relationship of care burden and motivation of COVID-19 vaccine acceptance. Given the aforementioned literature, it is of the utmost importance to investigate potential negative consequences of the care burden of those looking after those who have experienced strokes, to understand how to reduce COVID-19 vaccine hesitancy in the current unprecedented situation. According to recent findings, the Taiwanese population had a relatively low level of fear of COVID-19 and a low burden on stroke patients' caregivers [8]. This may be a result of the relatively mild extent of the COVID-19 outbreak in Taiwan. Taiwan has a high stroke incidence (330/100,000 annually) [26]. There was a decrease of approximately 13–16% in stroke admissions in Taiwan during the main COVID-19 outbreak period [26]. In the event of an outbreak of a highly contagious disease, timely and emergent acute stroke care may be compromised. In such challenging times, highlighting problems may provide important information for assessment and development of potential strategies to ensure adequate care for patients with acute or chronic stroke.

Therefore, the present study investigated the association of care burden with motivation of vaccine acceptance among caregivers of patients who have experienced stroke and explored the mediating role of problematic social media use, fear of COVID-19, and worry of infection in the relationship between care burden and motivation of COVID-19 vaccine acceptance. The study aimed to contribute to the existing scientific literature by testing the following exploratory hypotheses. It was expected that:

### H1

Care burden will negatively correlate with the motivation of vaccine acceptance.

### H2

Care burden will have indirect effects on worry of infection, fear of COVID-19, and vaccine acceptance through problematic social media use. More specifically, problematic social media use will act as a mediator between care burden and worry of infection (H_2a_), fear of COVID-19 (H_2b_), and motivation of vaccine acceptance (H_2c_).

### H3

The relationship between care burden and motivation of vaccine acceptance will be serially mediated by problematic social media use and worry of infection (H_3a_), and the relationship between care burden and motivation of vaccine acceptance will be serially mediated by problematic social media use and fear of COVID-19 (H_3b_).

## Methods

### Participants and procedure

The present study was cross-sectional in design. Participants were recruited from E-Da Hospital of Taiwan, a medical center that admits 1000 stroke patients every year. During 2021 in Taiwan, there was COVID-19 community outbreak during May to June. Moreover, by the end of June 2021, approximately 9% of individuals in Taiwan had received their first COVID-19 vaccination (based on Taiwan CDC data [27]). During the data collection stage in E-Da Hospital, there were less than 200 participants with COVID-19 infection per day in Taiwan. None of those enrolled participants in the present study had COVID-19 infection during data collection. The study included caregivers over 18 years who cared for stroke patients for more than four hours a day and who accompanied the patients who experienced a stroke during acute-phase and regular outpatient follow-ups. The caregiver was excluded if they (i) could not understand the survey items or (ii) had dementia, other cognitive impairment and/or severe hearing loss. Moreover, the majority family caregivers of patients with stroke were females, including patients’ wives, daughters, daughters-in-law, or granddaughters (~ 65%) [10, 28]. After consenting to participate in the study, all patients who had experienced a stroke completed neurological examination, including higher cortical functions, cranial nerves, motor, sensory and coordination tests. This was to ensure that the caregiver was caring for someone who had genuinely experienced a stroke. During inpatient or outpatient follow-up visits, research assistants gave caregivers verbal instructions and helped caregivers complete the survey. Regarding the data collection, the physician in the neurological clinic or ward reviewed the physical status and underlying diseases of those caregivers. Moreover, health education sheets were provided to clearly describe the benefit and possible side effects of COVID-19 vaccination. Furthermore, the research team evaluated the risk of potential thrombosis and embolism of vaccination for enrolled caregivers. The institutional review board (IRB) of E-Da Hospital approved the present study (Ref No. EMRP-110-079).

### Measures

#### Care burden

The Zarit Burden Interview (ZBI) was used in the present study. It contains 12 items rated on a five-point Likert scale (0 = not at all; 4 = extremely) to assess care burden of a caregiver. More specifically, the ZBI used in the present study was the shortened version developed by Ballesteros et al. [29]. A higher score of the ZBI indicates higher levels of care burden. The Chinese version of the 12-item ZBI used in the present study had been found to have good psychometric properties (Cronbach’s α = 0.84) [30, 31].

#### Problematic social media use

The Bergen Social Media Addiction Scale (BSMAS) was used in the present study. It contains six items rated on a five-point Likert scale (1 = very rarely; 5 = very often) to assess problematic social media use based on the components model of addiction [18]. A higher score of the BSMAS indicates a greater risk of problematic social media use. The BSMAS has been validated with promising good properties in many language versions [32–36], including the Chinese version used in the present study (α = 0.82) [37, 38].

#### Worry of infection

One self-developed item (“How much do you worry about being infected by COVID-19?”) was used to assess worry of infection. The item was rated using a Visual Analogue Scale from 1 (not at all worried) to 10 (extremely worried).

#### Fear of COVID-19

The Fear of COVID-19 Scale (FCV-19S) was used to assess fear of COVID-19. The FCV-19S consists of seven items rated a five-point Likert scale (1 = strongly disagree, 2 = disagree, 3 = neither disagree nor agree, 4 = agree, and 5 = strongly agree). The average score of the seven items was then computed to obtain the level of fear for a participant.

The present study used the Chinese version of the scale [39].

#### Motivation of vaccine acceptance

The Motors of COVID-19 Vaccine Acceptance Scale (MoVac-COVID19S; also known as the Drivers of COVID-19 Vaccine Acceptance Scale [DrVac-COVID19S]) was used in the present study [40]. It contains 12 items rated on a seven-point Likert scale (1 = strongly disagree; 7 = strongly agree) to assess motivation of COVID-19 vaccine acceptance. A higher score on the scale indicates higher levels of vaccine acceptance motivation. The MoVac-COVID19S has been validated with promising psychometric properties in many language versions [40–44], including the Chinese version used in the present study [45].

#### Participant characteristics

A background information sheet was used to collect information concerning the participants’ characteristics, including their age (in years), gender (male or female), care duration (in years), number of hours caring per day, relationship with the care recipient (spouse, son/daughter, or other), education (number of years), and marital status (currently married, single or other).

### Data analysis

The participants’ characteristics were firstly analyzed and summarized using descriptive statistics, such as means, standard deviations, frequencies, and percentages. Pearson correlations were used to examine the zero-order bivariate associations between the studied variables (care burden, problematic social media use, worry of infection, fear of COVID-19, and motivation of vaccine acceptance). Afterwards, several multiple linear regression models were constructed to further explore the associations between these variables with age, gender, care duration, and number of hours caring per day being controlled for. More specifically, the first regression model had care burden as the predictor with problematic social media use as the outcome; the second regression model had care burden and problematic social media use as predictors with worry of infection as the outcome; the third regression model had care burden and problematic social media use as predictors and fear of COVID-19 as the outcome; the fourth regression model had care burden, problematic social media use, worry of infection, and fear of COVID-19 as predictors and motivation of vaccine acceptance as the outcome.

Finally, Haye’s Process Macro [46] was used to examine the mediating effects of problematic social media use, worry of infection, and fear of COVID-19. All mediation models also controlled for age, gender, care duration, and number of hours caring per day. The Model 4 in the Process was firstly used to examine if problematic social media use was a significant mediator in the following associations: care burden to worry of infection, care burden to fear of COVID-19, and care burden to motivation of vaccine acceptance. Then, the Model 6 in the Process was used to examine if there were sequential mediating effects in the association between care burden and motivation of vaccine acceptance. More specifically, two mediation models with sequential mediation were constructed: (i) problematic social media use followed by worry of infection, and (ii) problematic social media use followed by fear of COVID-19. All the statistical analyses were performed using the IBM SPSS 20.0 (Armonk, NY: IBM Corp.).

## Results

Eight caregivers were not enrolled in this study (five declined to participate in the study and three could not speak Chinese). Therefore, the response rate was 95.6% (172/180). Among the 172 participants (mean age = 53.51 years [SD = 11.59]), less than one-third were males (n = 56; 32.6%). On average, they had contact with the care recipient 17.88 h per day (SD = 8.75), had cared for the care recipients for 2.81 years (SD = 4.12), and had received 10.92 years of education (SD = 4.17). Slightly more than one-third of the participants were the spouses (n = 65; 37.8%) or the sons/daughters (n = 65; 37.8%) of the care recipients. Just over three-quarters of the participants (n = 134) were currently married (77.9%) (Table 1).

**Table 1:** Characteristics of caregivers (N = 172)

Variables	Mean (SD)	n (%)
Age (in years)	53.51 (11.59)	
Gender (male)		56 (32.6)
Care duration (in years)	2.81 (4.12)	
Number of hours caring per day	17.88 (8.75)	
Relationship with the care recipient		
Spouse		65 (37.8)
Son/daughter		65 (37.8)
Other		21 (24.4)
Education (number of years)		10.92 (4.17)
Marital status		
Currently married		134 (77.9)
Single		15 (8.7)
Other		23 (13.4)

The correlations between the studied variables are presented in Table 2. Multiple regression results regarding how care burden, problematic social media use, worry of infection, and fear of COVID-19 associated with motivation of vaccine acceptance are presented in Table 3. After age, gender, care duration, and number of hours caring per day were controlled for, it was found that motivation of vaccine acceptance was significantly associated with care burden (standardized coefficient [β] = − 0.27;* p* = 0.004), problematic social media use (β = 0.35;* p* < 0.001), worry of infection (β = 0.31; *p* = 0.008), but not fear of COVID-19 (β = − 0.05; *p* = 0.66).

**Table 2:** Correlations between the studied variables (N = 172)

	*r* (*p*-value)				
	1	2	3	4	5
1. Care burden	–				
2. Problematic social media use	0.20 (0.008)	–			
3. Worry of infection	0.02 (0.81)	0.27 (0.001)	–		
4. Fear of COVID-19	0.21 (0.009)	0.28 (< 0.001)	0.68 (< 0.001)	–	
5. Motivation of vaccine acceptance	− 0.18 (0.02)	0.31 (< 0.001)	0.37 (< 0.001)	0.18 (0.02)	–

**Table 3:** Multiple linear regression models explaining vaccine attitudes (N = 172)

	Problematic social media use	Worry of infection	Fear of COVID-19	Motivation of vaccine acceptance
*B (p-value)*				
Age	− 0.11 (0.28)	0.004 (0.97)	− 0.09 (0.38)	0.05 (0.59)
Gender (Ref: female)	0.15 (0.09)	− 0.01 (0.89)	0.04 (0.70)	− 0.02 (0.83)
Care duration	− 0.11 (0.22)	− 0.11 (0.21)	− 0.04 (0.62)	− 0.05 (0.55)
Caring hours per day	− 0.08 (0.41)	0.12 (0.28)	0.18 (0.08)	0.07 (0.50)
Care burden	0.30 (0.001)	− 0.02 (0.81)	0.20 (0.03)	− 0.27 (0.004)
Problematic social media use	–	0.35 (< 0.001)	0.26 (0.006)	0.35 (< 0.001)
Worry of infection	–	–	–	0.31 (0.008)
Fear of COVID-19	–	–	–	− 0.05 (0.66)
*Fit statistics*				
F (*p*-value)	4.29 (0.001)	3.30 (0.005)	4.11 (0.001)	5.30 (< 0.001)
R^2^ (adj. R^2^)	0.150 (0.115)	0.147 (0.102)	0.175 (0.133)	0.273 (0.221)
VIF range	1.039–1.456	1.068–1.522	1.068–1.523	1.084–2.028

*B* Standardized coefficient, *VIF* Variance inflation factor

**Fig. 1:**
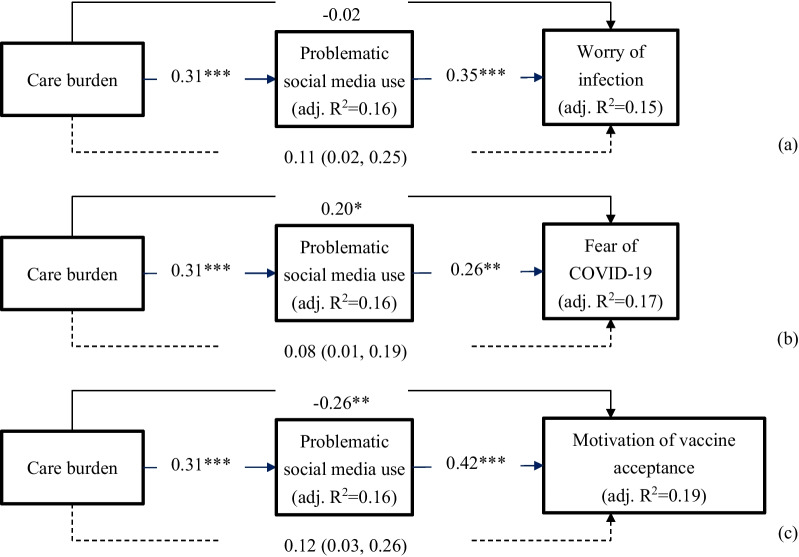
Mediating role of problematic social media use in the association of care burden with worry of infection, fear of COVID-19, and motivation of vaccine acceptance among caregivers of patients with stroke. Solid lines indicate direct effects with standardized coefficients; dashed lines indicate indirect effect with standardized coefficients (95% bootstrapping confidence interval). Age, gender, care duration, and number of hours caring per day were controlled in the models. *p < 0.05; **p < 0.01; ***p < 0.001

With regards to the mediation models, Fig. 1a shows that higher levels of care burden were associated with greater worry of infection via elevated problematic social media use (β = 0.11; 95% bootstrapping CI = 0.02, 0.25). However, care burden had no direct effect with worry of infection (β = − 0.02;* p* = 0.81). Figure 1b shows that higher levels of care burden were associated with greater fear of COVID-19 via elevated problematic social media use (β = 0.08; 95% bootstrapping CI = 0.01, 0.19). Moreover, care burden was positively associated with fear of COVID-19 (β = 0.20; *p* = 0.03). Figure 1c shows that higher levels of care burden were associated with greater motivation of vaccine acceptance via elevated problematic social media use (β = 0.12; 95% bootstrapping CI = 0.03, 0.26). Moreover, care burden was negatively associated with motivation of vaccine acceptance (β = − 0.26; *p* = 0.03). Moreover, adjusted R^2^ for problematic social media use was 0.16, 0.15 for worry of infection, 0.17 for fear of COVID-19, and 0.19 for motivation of vaccine acceptance.

**Fig. 2:**
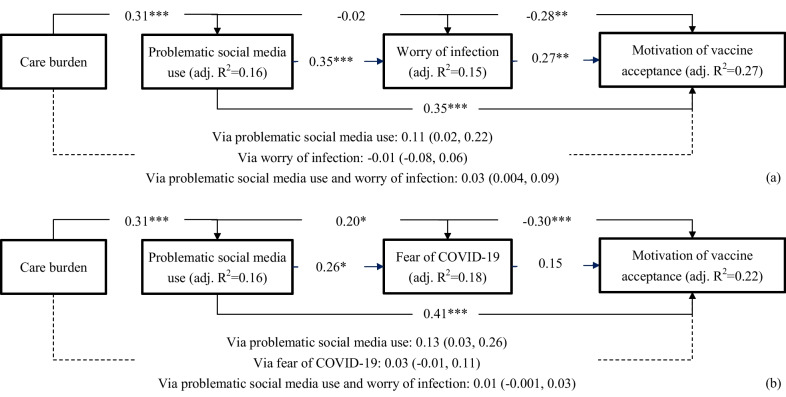
Mediating role of problematic social media use, worry of infection, and fear of COVID-19 in the association of care burden with motivation of vaccine acceptance among caregivers of patients with stroke. Solid lines indicate direct effects with standardized coefficients; dashed lines indicate indirect effect with standardized coefficients (95% bootstrapping confidence interval). Age, gender, care duration, and number of hours caring per day were controlled in the models. *p < 0.05; **p < 0.01; ***p < 0.001

Figure 2 shows that problematic social media use had sequential mediating effects together with worry of infection or fear of COVID-19 in the association between care burden and motivation of vaccine acceptance. Figure 2a shows that care burden was associated with motivation of vaccine acceptance via (i) problematic social media use alone (β = 0.11; 95% bootstrapping CI = 0.02, 0.22), and (ii) problematic social media use followed by worry of infection (β = 0.03; 95% bootstrapping CI = 0.004, 0.09), but not via worry of infection alone (β = − 0.01; 95% bootstrapping CI = − 0.08, 0.06). Figure 2b shows that care burden was associated with motivation of vaccine acceptance via problematic social media use alone (β = 0.13; 95% bootstrapping CI = 0.03, 0.26) but not the other two paths: β (95% bootstrapping CI) = 0.03 (− 0.01, 0.11) for fear of COVID-19 alone; β (95% bootstrapping CI) = 0.01 (− 0.001, 0.03) for problematic social media use followed by fear of COVID-19. The adjusted R^2^ was 0.16 for problematic social media use, 0.15 for worry of infection, 0.18 for fear of COVID-19, and 0.27 and 0.22 for motivation of vaccine acceptance. Moreover, the power of the two sequential mediation models was adequate (0.72 and 0.72).

## Discussion

The present study examined the association between care burden and motivation of COVID-19 vaccine acceptance, taking into the account the mediating role of problematic social media use, fear of COVID-19, and worry of infection among caregivers of patients who had experienced a stroke. The analysis results found the direct association between care burden and motivation of COVID-19 vaccine acceptance, indicating that as care burden increased for caregivers who took care of patients who had experienced a stroke, their motivation to get COVID-19 vaccinated decreased (H_1_ supported). This might be due to stress the caregivers faced when making healthcare decisions for themselves while they have to provide care to patients who have experienced a stroke without formal services being available during the pandemic. Therefore, tailored awareness programs are needed to inform caregivers to take appropriate health decisions for themselves, which can further support them to provide quality care to patients who have experienced a stroke. That is, there is a need to increase caregivers’ awareness of the need for the COVID-19 vaccine to be taken.

Problematic social media use positively mediated the relationship between care burden and worry of infection (H_2a_ supported), as well as the relationship between care burden and fear of COVID-19 (H_2b_ supported). Therefore, when caregivers experience stress and strain due to high care burdens, they may use social media maladaptively, causing them fear and worry concerning COVID-19 infection. Previous research has shown separate positive associations between relationship of (i) care burden and problematic social media use [47], and (ii) problematic social media use and fear of COVID-19 [48]. Consequently, the present study advanced knowledge of these associations by demonstrating how problematic social media use mediates these relationships.

In addition, the present study findings showed that despite increasing care burden being associated with decreasing motivation of COVID-19 vaccine acceptance, the mediating effect of problematic social media use was positively associated with this relationship (H_2c_ supported). This finding suggests that high care burden among caregivers with problematic social media use might be less COVID-19 vaccine hesitant. Additionally, it might be that this group of individuals is more open to utilizing social media in helping them make their care recipient health-related decisions [49]. Although problematic social media use seemed to increase caregivers’ motivation to get COVID-19 vaccinated, cautious should be made because problematic social media use may contribute to psychological distress [25]. Indeed, the present study found that problematic social media use was associated with fear and worries. A proactive approach is therefore needed from health experts and practitioner in disseminating accurate and trustworthy factual information regarding COVID-19 with mental health supporting programs to help caregivers cope with psychological distress this may also reduce the psychological distress associated with social media use, while maintaining their motivation of COVID-19 vaccine acceptance.

The study’s mediation analysis also showed that care burden was associated with motivation of vaccine acceptance through problematic social media use followed by worry of infection (H_3a_ supported) and fear of COVID-19 (H_3b_ supported). This finding suggests that caregiver's overreliance on social media for information during a pandemic might (for some) result in problematic social media use, which in turn could lead to worry about infection due to news circulating about COVID-19 on social media platforms. Subsequently, this leads to an increase in motivation of vaccine acceptance. Previous studies have shown that, worrying about infection leads to increase in COVID-19 vaccine acceptance [24], whereas a previous study by Ahorsu et al. [48] showed no direct association between problematic social media use and intention to get a COVID-19 vaccine. Nevertheless, the present study advances knowledge in this area by extending the evidence to the cohort of caregivers of patients who have experienced a stroke, and explaining the indirect association of care burden and motivation of COVID-19 vaccine acceptance. Therefore, social media appears to play an important role to increase motivation of vaccine acceptance among caregivers of vulnerable populations by providing relevant information on the spread of infection and the worrisome situation due to COVID-19 infection. Based on the aforementioned literature, it appears that the total variances in motivation of vaccine acceptance explained by care burden, problematic social media use, worry of infection, and fear of COVID-19 were modest (i.e., 22–27%).

There are some limitations to the present study. First, the cross-sectional design cannot provide any causal evidence regarding the relationships between the variables studied. Second, the present study's convenience sampling method from a single Taiwanese hospital with a small sample limits its representativeness and generalizability to other Taiwanese caregiving citizens. Third, as the participants were all recruited in Taiwan, the findings might not be entirely applicable to caregivers in non-Asian countries without collectivist backgrounds. Fourth, some conditions of the patients who have experienced a stroke were not assessed in the present study (e.g., limitations in daily activities; dependency levels on everyday activities). Conditions like this may affect caregiver burden and serve as confounders in the present study. Therefore, it is necessary to conduct longitudinal studies among more representative samples from both in and outside of Taiwan in to address the aforementioned limitations.

## Conclusion

The present study found a direct association between care burden among caregivers who took care of patients who had experienced stroke and motivation of COVID-19 vaccine acceptance. The present study also found several indirect (mediating) associations in the aforementioned association via problematic social media use, as well as problematic social media use followed by worry of infection. This implies that increased care burden among stroke caregivers can lead to decrease in COVID-19 vaccine acceptance. However, problematic social media use seemed to increase caregivers’ motivation to get COVID-19 vaccinated, and this may due to the information they obtained from the social media. Although the problematic social media use increased caregivers’ motivation to get COVID-19 vaccinated, it also increased their psychological distress. Therefore, support for the caregivers is urgently required to mitigate their caregiving burdens during the pandemic (and future pandemics) and to increase COVID-19 vaccine acceptance by providing relevant and accurate information on social media platforms without elevating their psychological distress. Given that kinship networks play an important role in caregiving support in Taiwan, the study had potential limitations regarding its generalizability. Therefore, longitudinal studies are needed to determine more clearly the causal relationships between the study variables and whether the present study's findings are applicable to other regions or cultures with different caregiving norms or support systems.

## Data Availability

The data that support the findings of the present study are available from the corresponding author upon reasonable request.
